# A simplified, data-constrained approach to estimate the permafrost carbon–climate feedback

**DOI:** 10.1098/rsta.2014.0423

**Published:** 2015-11-13

**Authors:** C. D. Koven, E. A. G. Schuur, C. Schädel, T. J. Bohn, E. J. Burke, G. Chen, X. Chen, P. Ciais, G. Grosse, J. W. Harden, D. J. Hayes, G. Hugelius, E. E. Jafarov, G. Krinner, P. Kuhry, D. M. Lawrence, A. H. MacDougall, S. S. Marchenko, A. D. McGuire, S. M. Natali, D. J. Nicolsky, D. Olefeldt, S. Peng, V. E. Romanovsky, K. M. Schaefer, J. Strauss, C. C. Treat, M. Turetsky

**Affiliations:** 1 Earth Sciences Division, Lawrence Berkeley National Lab, Berkeley, CA, USA; 2 Center for Ecosystem Science and Society, Northern Arizona University, Flagstaff, AZ, USA; 3 Department of Civil and Environmental Engineering, University of Washington, Seattle, WA, USA; 4 School of Earth and Space Exploration, Arizona State University, Tempe, AZ, USA; 5 Met Office Hadley Centre, Exeter, UK; 6 Environmental Sciences Division, Oak Ridge National Laboratory, Oak Ridge, TN, USA; 7 Laboratoire des Sciences du Climat et de l’Environnement (LSCE CEA-CNRS-UVSQ), Gif-sur-Yvette, France; 8 Alfred Wegener Institute Helmholtz Centre for Polar and Marine Research, Periglacial Research Unit, Potsdam, Germany; 9 United States Geological Survey, Menlo Park, CA, USA; 10 Department of Physical Geography, Bolin Centre of Climate Research, Stockholm University, Stockholm, Sweden; 11 National Snow and Ice Data Center, University of Colorado, Boulder, CO, USA; 12 Laboratoire de Glaciologie et Géophysique de l’Environnement, CNRS and Université Grenoble Alpes, Grenoble 38041, France; 13 Climate and Global Dynamics Division, National Center for Atmospheric Research, Boulder, CO, USA; 14 School of Earth and Ocean Sciences, University of Victoria, Victoria, British Columbia, Canada; 15 Geophysical Institute Permafrost Laboratory, University of Alaska, Fairbanks, AK, USA; 16 US Geological Survey, Alaska Cooperative Fish and Wildlife Research Unit, University of Alaska Fairbanks, Fairbanks, AK, USA; 17 Woods Hole Research Center, Falmouth, MA, USA; 18 Department of Renewable Resources, University of Alberta, Edmonton, Alberta, Canada; 19 Department of Integrative Biology, University of Ontario, Guelph, Ontario, Canada

**Keywords:** permafrost, climate change, carbon–climate feedbacks, methane

## Abstract

We present an approach to estimate the feedback from large-scale thawing of permafrost soils using a simplified, data-constrained model that combines three elements: soil carbon (C) maps and profiles to identify the distribution and type of C in permafrost soils; incubation experiments to quantify the rates of C lost after thaw; and models of soil thermal dynamics in response to climate warming. We call the approach the Permafrost Carbon Network Incubation–Panarctic Thermal scaling approach (PInc-PanTher). The approach assumes that C stocks do not decompose at all when frozen, but once thawed follow set decomposition trajectories as a function of soil temperature. The trajectories are determined according to a three-pool decomposition model fitted to incubation data using parameters specific to soil horizon types. We calculate litterfall C inputs required to maintain steady-state C balance for the current climate, and hold those inputs constant. Soil temperatures are taken from the soil thermal modules of ecosystem model simulations forced by a common set of future climate change anomalies under two warming scenarios over the period 2010 to 2100. Under a medium warming scenario (RCP4.5), the approach projects permafrost soil C losses of 12.2–33.4 Pg C; under a high warming scenario (RCP8.5), the approach projects C losses of 27.9–112.6 Pg C. Projected C losses are roughly linearly proportional to global temperature changes across the two scenarios. These results indicate a global sensitivity of frozen soil C to climate change (*γ* sensitivity) of −14 to −19 Pg C °C^−1^ on a 100 year time scale. For CH_4_ emissions, our approach assumes a fixed saturated area and that increases in CH_4_ emissions are related to increased heterotrophic respiration in anoxic soil, yielding CH_4_ emission increases of 7% and 35% for the RCP4.5 and RCP8.5 scenarios, respectively, which add an additional greenhouse gas forcing of approximately 10–18%. The simplified approach presented here neglects many important processes that may amplify or mitigate C release from permafrost soils, but serves as a data-constrained estimate on the forced, large-scale permafrost C response to warming.

## 1. Introduction

Carbon (C) cycle feedbacks to climate change remain a large uncertainty in our ability to project climate change originating from anthropogenic greenhouse gas emissions. Because ecosystems may either gain or lose C in response to warming that is caused by C emissions, these ecosystem C changes may act to amplify or mitigate this warming. The magnitude of this amplification or mitigation can be approximated as a linear feedback term, *γ*, defined as the amount of global C change of a given system per unit of global temperature change. There are many such feedbacks in the Earth system, which combine to determine the overall sensitivity of the planet to climate perturbations [1–3]. Early attempts to quantify terrestrial C feedbacks using process-based Earth system models (ESMs) tended to focus on temperate and tropical regions, where C stocks are mainly in vegetation and surface soils [4,5]. However, large stocks of C are buried in soils at high latitudes [6,7], with recent estimates of the magnitude of the permafrost C stocks estimated at 1035±150 Pg C in soils to 3 m depth [8] plus 213±41 [9] to 456±45 Pg C [10] in deeper Yedoma and thermokarst deposits that have formed over the period of thousands to tens of thousands of years ago, for a total of 1330–1580 Pg C [11]. These deep deposits represent the single largest organic C pool in terrestrial ecosystems, and are to a large extent stabilized by being frozen and/or waterlogged, which in both cases are highly climate-dependent. It has therefore become increasingly clear that the potential feedback effects from warming northern soils must be more accurately included in estimates of terrestrial C cycle feedbacks. Initial attempts to include a set of processes governing permafrost C cycling have now been included in a set of terrestrial C cycle models and ESMs (reviewed in [11,12]), which give estimates of C release by 2100 because of thawing permafrost in the range of 37–174 Pg C. The last Intergovernmental Panel on Climate Change report [13] assessed a similar although larger range of 50–250 Pg C, based on simplified model estimates [14,15] before the two above review studies were published. This potential response is large enough to be a globally relevant contribution to the overall climate response to human greenhouse gas emissions.

There is reason to believe that larger amounts of warming will lead to larger permafrost C responses; however, the model studies reviewed in [11,12] have not all reported the global temperature changes that were used to drive the permafrost responses. It is therefore not possible to infer the sensitivity of projected C losses from these process-based models to the amount of warming, a crucial step in understanding the magnitude of the permafrost carbon–climate feedback, *γ*_P_. It is also unclear whether the assumption of linearity implicit in the concept of the *γ*_P_ feedback parameter is valid for permafrost soils, or whether instead there are global temperature thresholds in the response of these soils to warming, or if permafrost C could continue to lose C even if warming is stabilized in the future. Estimates of *γ*_P_ from simple models based on CMIP5 model soil temperatures show a wide range of values [13,15]. Because terrestrial C cycle feedbacks represent a large source of uncertainty on the relationship between anthropogenic C emissions and global temperature change, and because permafrost soils may constitute an important but widely overlooked component of these feedbacks, it is imperative to better quantify the magnitude of *γ*_P_, and to determine whether or not such temperature thresholds exist, so that we can incorporate these processes into estimates of Earth’s overall climate sensitivity to greenhouse gas emissions.

Considerable progress has been made in recent years to synthesize datasets needed to quantify the magnitude of expected C release from permafrost soils, including the stock estimates described above, the vertical distribution and characteristics of C in different permafrost soil types [16] and the dynamics of decomposition under aerobic and anaerobic incubation conditions [17–19]. Furthermore, there has been an effort to improve the representation of soil and snow physical processes that determine the thermal properties of permafrost soils in terrestrial models, which were poorly represented in the CMIP5 generation of ESMs [20].

Many of these pieces of information can be assembled, using a simplified model, to provide a data-constrained estimate of the magnitude of the permafrost carbon–climate feedback. Our goal in this paper is to construct such a model and use it to estimate one aspect of the permafrost carbon–climate feedback term: that related to the enhanced decomposition arising from warmer soils and thawing permafrost. While this large-scale warming and thawing represents only one possible avenue for C losses from permafrost soils, this simplified scaling approach may serve as a useful quantification of the potential magnitude of a major component of the feedback, and serve as a comparison with more complex model representations of carbon–climate feedback effects from high-latitude soils.

## 2. Methods

### (a) Overall approach

To construct a simplified model of permafrost C cycle dynamics that is as closely tied to observations as possible, we base our approach on three main components, each of which has been synthesized as part of activities organized by the Permafrost Carbon Network (PCN), (www.permafrostcarbon.org). The first component is to start with recently compiled soil C maps. Terrestrial C cycle models typically are initialized with soil C distributions that are calculated by finding a steady-state condition where soil C inputs equal losses, and these steady-state C maps are generally not an accurate representation of actual C stocks [21]. In particular, processes unique to permafrost soils such as freeze–thaw mixing and the syngenetic freezing of lower soil layers with continuing sedimentation are typically not represented in C cycle models, nor are they initialized over a sufficiently long period and with glacial climate conditions to include soil C that has been deposited over the Pleistocene. To get around these limitations in C cycle models, our approach is to initialize our simple model with observations of soil C that have been upscaled using thematic soil classification maps [8] as the initial state of the permafrost C cycle calculation. We do still use an initial steady-state assumption, as discussed further below, but instead of letting soil C adjust until inputs match losses, we set inputs to match inferred initial losses given known C stocks and turnover times.

The second component of our model is to use the laboratory incubation syntheses as the basis for the transient dynamics of permafrost soil C losses resulting from microbial decomposition of soil organic matter (SOM). Typical terrestrial C models use a single set of global decomposition constants, such as inherent SOM pool turnover times, C use efficiencies or the fractional partitioning into each pool. Here, we use a recent synthesis of incubation dynamics from permafrost soils [17] to build a simplified decomposition model of the soil horizon types being subjected to thaw, i.e. conceptually similar to the C module of complex C cycle models. While there are many uncertainties and assumptions in such an approach, it has the advantage that it is more highly constrained by data specifically from permafrost soils than the standard approach used in C cycle models.

The third key element is to force the incubation-derived decomposition rates with multi-model predictions of soil temperatures in response to an imposed climatic warming over this century. Putting the three elements of our simple model together, we call this the PCN Incubation–Panarctic Thermal (PInc-PanTher) scaling approach. Where large uncertainties exist in the driving data of this approach, we have tried to bracket the possible range of responses using different values for those parameters. Table 1 outlines key aspects of the PInc-PanTher approach and how it differs from traditional ecosystem modelling approaches.

**Table 1.** Overview of key processes in PInc-PanTher and how they differ from representation in full ecosystem models.

ecosystem property	PInc-PanTher	ecosystem model
initial soil C content geographical distributions	set directly from upscaled soil classification maps	calculated to satisfy initial condition that C losses balance inputs
initial soil C content vertical distributions	set directly from a combination of upscaled soil classification maps and soil C vertical profile synthesis	either ignored or calculated assuming a vertical distribution to C inputs and vertical transport
soil C inputs	calculated to satisfy initial condition that soil C losses balance inputs, and held fixed in time	calculated based on routing vegetation productivity to soil pools; vary because of changes in plant productivity
soil C pool distribution	set to correspond to simple three-pool fitted to permafrost incubation data, and specific to soil horizon types	fraction of C from a given plant organ to a given litter or SOM pool in decomposition pathway fixed globally or vary by plant functional type
temperature control of decomposition	*Q*_10_ function, truncated to stop respiration when soil is frozen	diverse temperature functions, typically *Q*_10_, Arrhenius, or similar; freeze effects may be included via soil moisture term
other environmental controls on decomposition	anoxia prescribed for all peat (Histel) soils	may include limitations by anoxia, soil moisture, depth, nutrients
soil temperatures	imposed based on thermal modules of ecosystem models used to drive PInc-PanTher	calculated dynamically based on atmospheric climate that is either imposed (offline ecosystem model) or calculated in climate model (ESM)
CH_4_ dynamics	emissions held as a constant fraction of heterotrophic respiration from anoxic soils, which are assumed to correspond to peat (Histel) soils	typically treat CH_4_ production, transport and oxidation for separate flooded and unflooded gridcell fractions

### (b) Estimates of C stocks

**Figure 1.**
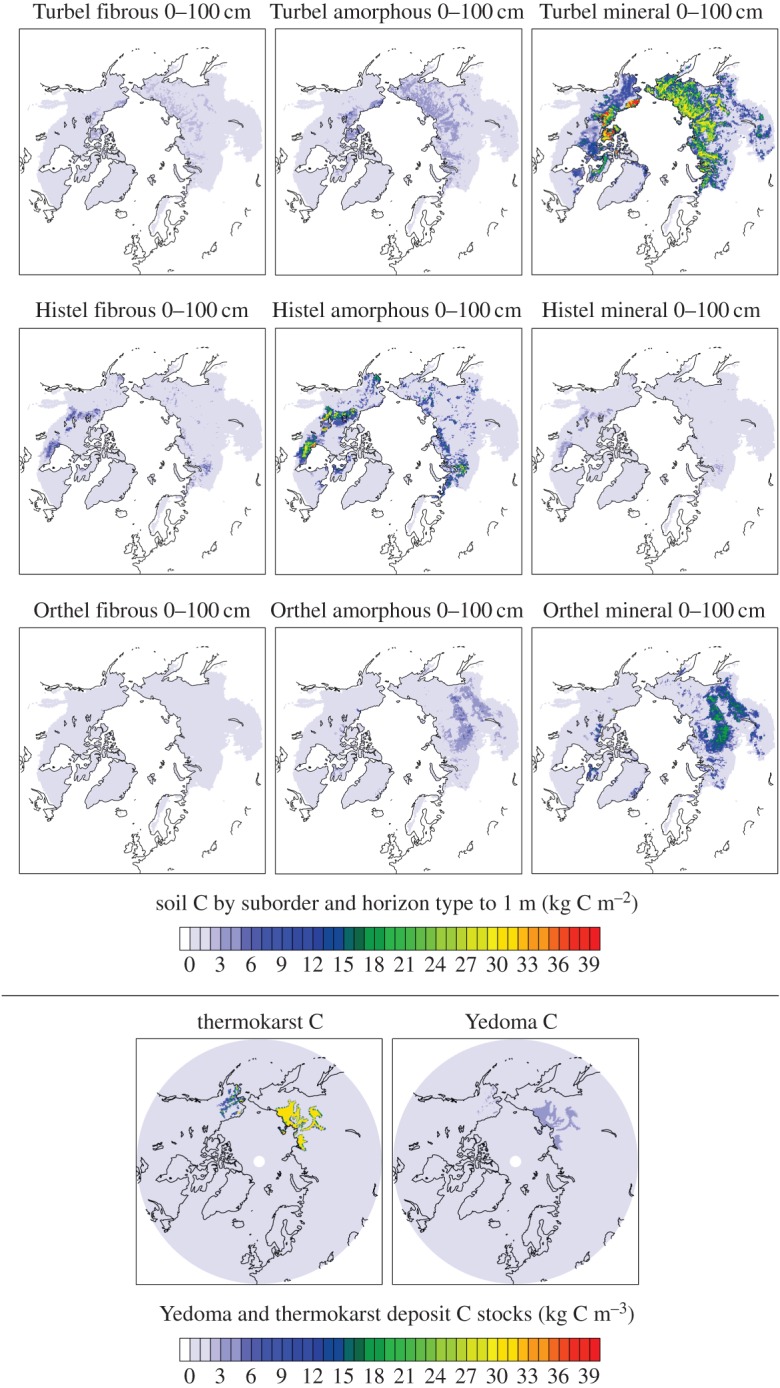
Maps of soil C by horizon type and suborder for 0–100 cm intervals, as well as deeper Yedoma and thermokarst deposits.

For soil C stocks, we use the panarctic permafrost soil C maps described by Hugelius *et al*. [8] for surface (0–3 m) soils. All estimates use only the soil C from the three Gelisol suborders (Histel, or permafrost soils with thick (greater than 40 cm), peaty organic layers; Turbel, or permafrost soils showing evidence of cryoturbation; and Orthel, or permafrost soils that show neither thick organic layers nor cryoturbation). In addition to disaggregating the soils by suborder, we also disaggregate the soil C by horizon type into three groups: fibrous organic horizon (e.g. peaty horizons), amorphous organic horizons (finely dispersed organic matter) and mineral horizons. This partitioning is based on the soil C profiles compiled by Harden *et al*. [16], in which soil C was partitioned into horizon types for each of the three Gelisol suborders as functions of depth. Disaggregated soil C maps to 1 m are shown in figure 1. Deeper soil layers show similar patterns, with the bulk of C in mineral horizons for Turbel and Orthel soils, and in amorphous horizons for Histel soils. Overall C contents follow the maps shown in Hugelius *et al*. [8].

To correspond to the soil thermal models, soil C maps at 0–30 cm, 0–1 m, 1–2 m and 2–3 m were first rasterized to 0.25 degree resolution, then regridded using a mass-conservative interpolation to the geographical grids of each of the soil thermal models, then regridded vertically, again using a mass-conservative interpolation, to the vertical grid of each soil thermal model. Total integrated C stocks of the 0–3 m soils after all regridding were within ±0.5% of the 727 Pg C for all Gelisol soils reported in Hugelius *et al*. [8].

Note that we do not include dynamics of non-permafrost soil orders occurring within discontinuous permafrost regions, which comprise another 280 Pg C in the circum-boreal permafrost region [8]. The reason for this is that we are primarily interested in calculating the response due to the deepening of the active layer, loss of permafrost, warming of soils and lengthening of seasonal thaw period. As these soils are estimated to not initially have permafrost present, the effect of permafrost loss does not make sense for them. Nonetheless they will probably play a role in C cycle changes as they will also be subject to enhanced decomposition through longer thawed season length [22], but their role as potential C cycle responses may be more amenable to estimation using the traditional single-layer soil models contained in the current generation of ESMs.

We also include estimates of permafrost C below 3 m depth, using the Strauss *et al*. [9] compilation of perennially frozen Yedoma and thermokarst deposits in the large Yedoma region of East Siberia and Alaska. Yedoma deposits are ice-rich polygenetic sediments dominated by alluvial and aeolian deposits and syngenetic ground ice that formed during the Late Pleistocene in unglaciated Arctic lowlands and foothills [23,24]. Yedoma deposits can have large amounts of undecomposed C frozen in them [25], and because of large ice-wedges and excess pore ice content, these kinds of deposits are prone to deep thaw [26]. Thermokarst deposits formed following the degradation of icy Yedoma deposits by lakes during the Holocene. The formation of lakes was associated with melting of volumes of segregated ground ice, and resulted in ground surface subsidence (a process known as thermokarst), deep thaw of permafrost underneath the lakes (so-called talik) and subsequent accumulation of lake sediments in the basins. Rapid thaw in these regions then frequently resulted in drainage of thermokarst lakes, exposing lake sediments and unfrozen taliks to Arctic subaerial conditions under which the deposits refroze.

Strauss *et al.* [9] show two different estimates of Yedoma areal extent. The first uses the newer more detailed but geographically incomplete map from Grosse *et al*. [27] for Yedoma and thermokarst deposits in Siberia; the second uses the older but more geographically complete map of potential Yedoma deposits [28]. For Alaskan Yedoma [9], we use the map of Jorgenson *et al*. [29] in both cases. Here, we use the Romanovskii [28] estimate of potential Yedoma, but scaled to remove areas that have been through a Holocene thermokarst cycle as estimated by Grosse *et al*. [27].

For C thickness and C density in the Yedoma region deposits, we use the estimates of C from Strauss *et al*. [9], who report mean C concentration of 14 kg C m^−3^ for Yedoma deposits and 56 kg C m^−3^ for thermokarst deposits, and a mean depth of 19.4 m for Yedoma and 5.5 m for thermokarst. Walter Anthony *et al.* [10] show that an additional 114 Pg is stored in deep taberites (diagenetically altered Yedoma deposits) underlying Holocene thermokarst deposits and lakes, but these are not included here as portions of this pool underneath lake bottoms are still thawed and will therefore have a different trajectory with warming from that for permafrost soils or deposits. Yedoma stocks using the Romanovskii [28] map are scaled by a factor of 0.3 to account for Holocene losses to thermokarst [9], and the remaining 0.7 of the area is treated as thermokarst, but multiplied by a factor of 0.8 to remove lake-covered areas [9]. The upper 3 m in all cases are treated as soils and use the soil C concentrations derived from Hugelius *et al*. [8] rather than the deeper Yedoma and thermokarst concentrations.

The estimates of deep Yedoma and thermokarst are also shown in figure 1. Note that the mean estimates reported in Strauss *et al*. [9] are the larger of the two estimates provided, with median-based estimates reported at 10 kg C m^−3^ (Yedoma) and 31 kg C m^−3^ (thermokarst). As we show below, the actual contribution of these deep C stocks to the projected carbon–climate feedback are small compared with the surface soils because we do not include fine-scale ice melting and ground subsidence processes, which may lead to more rapid thaw and erosion of permafrost, in the model. Thus these estimates can be considered an upper limit on the direct C response of these deep deposits (excluding taberites) to large-scale, gradual soil thaw, but a lower limit on their total response that would additionally be affected by fine-scale, rapid thaw processes.

### (c) Estimates of C decomposability and dynamics

We base our estimates of C decomposition rates on the aerobic soil incubation meta-analysis of Schädel *et al*. [17], in which data from a set of incubations were fitted to a parallel three-pool, first-order decomposition model to calculate a set of parameters that best describe permafrost C losses for these incubations. Schädel *et al*. [17] describe two main methods for partitioning the variance between different soil samples: (i) they use the C : N ratio of each soil sample to derive a regression relationship between the fraction of C in pools with different turnover times and the soil C : N ratio and (ii) they separately determine decomposition parameters for organic and mineral horizons, where organic horizons are defined as those having more than 20% C by mass and mineral horizons are those that have less than 20% C. We use both methods to better assess the uncertainty in C projections arising from the uncertainty in decomposition parameters.

Decomposition in the PInc-PanTher approach is treated as a series of three exponential terms, corresponding to an active, slow and passive pool. The turnover times of the individual pools at the reference temperature *T*_*ref*_ of 5°C are (i) for organic horizons (fibrous and amorphous horizons of figure 1): 0.41 years, 7.21 years and 125 years; (ii) for mineral horizons: 0.48 years, 8.76 years and 2500 years [17]. These are different from the actual ages of the pools as they describe the dynamics of soils once thawed in a laboratory rather than frozen in permafrost. Because the incubations, while long for such experiments, were much shorter than the actual turnover times of the passive pools, the passive pool turnover times were specified *a priori* rather than inferred from the incubations, so that the meta-analysis inferred only the turnover times of the fast and slow pools, and the fraction of C in the fast and slow pools (and, by difference, the fraction of C in the passive pools) [17].

We need to know the initial partitioning of C stocks among these pools (which is not provided in the soil C stocks datasets). To estimate the initial partitioning, for the C : N ratio method, we use the C : N ratios reported for fibrous, amorphous and mineral horizons in Harden *et al*. [16] (which are 39.1, 25.1 and 17.3, respectively) to infer the pool partitioning based on the Schädel *et al*. [17] regression relationship. Schädel *et al*. [17] report that the pool fractions can be estimated linearly: *C*_fast_=10^(0.006RC:N−0.15)^/100; 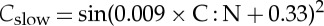; *C*_passive_=(1−(*C*_fast_+*C*_slow_)). This approach gives initial pool partitioning of 1.25%, 40% and 59% for fibrous (each percentage corresponds to active, slow, and passive, respectively); 1.0%, 28% and 72% for amorphous; and 0.9%, 22% and 77% for mineral horizons. Uncertainty ranges calculated via 95% confidence intervals around the Schädel *et al*. [17] relationship of the fast, slow and passive pool fractions are: for fibrous, 0.32–2.9%, 20–74% and 23–80%; for amorphous, 0.24–2.5%, 14–56% and 42–86%; and for mineral, 0.2–2.3%, 11–45% and 52–89%. For the second partitioning method, we use only the mean pool partitioning values of the organic and mineral horizons from Schädel *et al*. [17]. This approach gives initial pool partitioning of 1.5%, 29% and 69% for both amorphous and fibrous organic horizons; and 1.0%, 13% and 87% for shallow mineral horizons, which are all within the large uncertainty ranges of estimates derived using the C : N partitioning. We note that the uncertainty on this pool partitioning is high, and that fully propagating the entire range of this uncertainty would lead to an even larger range of uncertainties than reported here.

To project the effect of changing soil temperatures on decomposition rates, we use a truncated *Q*_10_ function. At or below the freezing point, we assume zero respiration. Above the freezing point, we assume that respiration rates follow an exponential curve with a *Q*_10_ of 2.5 [17], i.e. that respiration rates increase by a factor of 2.5 for each 10°C increase in soil temperature.

The assumption of zero respiration in frozen soil layers implies that no decomposition occurs initially in permafrost layers; however, decomposition does proceed in the current climate in the active layer, which is the upper layer of soils above permafrost that thaws during the summer and completely refreezes during the following winter. We are interested in a simplified model approach for projecting soil C losses in response to soil warming throughout the soil column, and therefore need to remove the effect of decomposition that would occur even under a constant climate. As discussed above, terrestrial C cycle models have traditionally done this by making an assumption of initial C balance from a steady-state spin-up of their C pools (that may not represent old permafrost C), and then finding the set of C stocks that allow for this steady-state condition to exist given a set of inputs by productivity and outputs by respiration. Here, we adapt this assumption to the problem of known initial C stocks and decomposition rates in order to find the set of inputs to each C pool at every location that satisfies the initial steady-state assumption by solving the equation
2.1
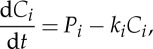
where *C*_*i*_ is the initial C stock of pool *i*, *P*_*i*_ are the inputs to pool *i* and *k*_*i*_ is the decay constant (equal to 1/*τ*_*i*_, the turnover time), to find the inputs to each pool required to meet the steady-state condition d*C*_*i*_/d*t*=0,

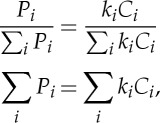
for the fractional and total inputs into each pool. Note that the *k* values above are functions of soil temperature and so differ from the incubation-derived decomposition constants via the temperature function; in particular, both initial *k* values and therefore inputs are zero for permafrost soil layers. Because the total inputs, integrated vertically and summed over each pool and horizon type, give an estimate of the net litterfall input required to maintain initial steady state, we can use this as a rough test of the model realism, subject to the caveats discussed below. There are of course limits to the assumption of steady state in the models, and these limits are particularly true for high-latitude soils where the time scales required to reach equilibration are quite long; nonetheless, this approach allows for us to ask how soil C may change as a result of warming, given a set of constant inputs and holding all else constant, relative to what changes would occur in the absence of that warming.

Many soils in the permafrost-affected region are both cold and wet, and the anoxia and fire protection that results from this wetness is a key reason why certain soils, in particular peat soils, are so high in C. Thus, it would be incorrect to assume that only temperature limits decomposition throughout the permafrost region, and we therefore must include a process by which decomposition is also slowed by anoxia. For this analysis, we are interested in how warming will influence soil C stocks holding all else constant, and so we would like to also calculate the greenhouse gas response from wet soils that warm but remain anoxic following thaw.

There is high uncertainty on the distribution of flooded or otherwise anoxic conditions in the permafrost region; as one possible estimate of anoxic soils, we use the soil C maps and assume that all Histel (permafrost-affected peat, which covers 1.4 million km^2^, or 13% of total Gelisol area [8]) soils are fully saturated and remain so under warming, and apply this anoxic reduction term to all decomposition of the Histel soils. There are limitations to this assumption: for example, Histel soils can be dry in peat plateaus, and Turbel or Orthel soils can be wet for at least a fraction of the year, and, in all cases, the saturation is likely to change with warming, but it serves as a rough starting point for this approach.

To estimate decomposition rates of anoxic soils after thawing, we use a meta-analysis of paired oxic and anoxic incubations [18]. In this meta-analysis, the ratio of C release (sum of both CO_2_ and CH_4_ production) under oxic versus anoxic incubation conditions was calculated using nine different incubation studies with soils from the permafrost zone. The ratio of C release was consistently 3.4 times higher when the same unit of soil was incubated under oxic versus anoxic conditions with no detectable control of this ratio by incubation temperature, soil C concentration, vegetation type and frozen state (active layer or permafrost).

### (d) CH_4_ emissions

The total C release from anoxic soils comprises both CO_2_ and CH_4_. To calculate potential CH_4_ emissions in addition to the CO_2_ losses, we assume that a constant fraction of these anoxic C losses are emitted as CH_4_ to the atmosphere. In reality, CH_4_ dynamics are more complicated than CO_2_ dynamics, with large production and consumption of CH_4_ within soils, large differences in the CH_4_ production rates of different soils based on biome and geomorphology [19], and complex dynamics of fine-scale and seasonally varying water table dynamics [30], leading to changes in the CO_2_:CH_4_ ratio of surface fluxes, such that process models of wetland CH_4_ emissions show poor agreement in their response to forcings [31]. Nonetheless, we are interested in understanding the potential response of just the forced, large-scale warming of soils and consequent increase in respiration that may drive increased CH_4_ emissions, in the absence of changes to inundation, plant inputs, water table depth, soil CH_4_ transport efficiency, etc. Here incubations are less useful as a guide for scaling respiration rates to CH_4_ fluxes, as anoxic incubation necessarily does not include methanotrophic consumption of CH_4_ in oxic soil layers during transport to the surface, and, therefore, would require a poorly constrained scaling factor to relate large-scale anoxic respiration rates to CH_4_ fluxes.

To avoid dependence on this scaling factor, we report below the fractional change to anoxic respiration, i.e. the integrated anoxic respiration divided by the initial integrated anoxic respiration averaged over the first 10 years of the scenario. Then we assume that warming-induced changes to CH_4_ fluxes are proportional to the change in anoxic respiration, and put this change in the context of the current permafrost-zone wetland CH_4_ emissions. The reason for assuming that CH_4_ flux changes are proportional to anoxic respiration changes rather than anoxic soil C changes is that plant inputs account for a large fraction of the substrate fuelling CH_4_ emissions [32]; because we include these plant fluxes as inputs to the decomposition model, most of which turn over quickly, they constitute a large fraction of the respiration flux. Instead of using the CH_4_ production rates from anoxic incubations as the basis for calculating CH_4_ emissions due to the differences between methane production and flux and the fact that incubations exclude the plant C inputs, we can use top-down estimates of current CH_4_ fluxes [33] as the absolute flux against which the proportional change in CH_4_ emissions is applied. Finally, we use the resulting absolute changes in CH_4_ from the top-down approach to compare the CO_2_:CH_4_ flux ratios inferred for anoxic soils in the scaling approach to incubation CO_2_:CH_4_ ratios as an independent though imperfectly corresponding constraint on the validity of the approach.

Note that this approach for calculating CH_4_ emissions differs from the more typical method of multiplying CH_4_ flux density and wetland extent (e.g. [31,34]). Our calculation does not to first order depend on the areal extent of wetlands; the uncertainty in this term is already included in the use of integrated fluxes from Kirschke *et al*. [33] as our background flux, which we scale in proportion to the fractional change over time of respiration. Instead the main purpose of using the Histel areal fraction is as a geographical weighting to identify the warming-induced response from regions most responsible for CH_4_ fluxes.

### (e) Estimates of soil temperature and response to climate change

The last required component of the PInc-PanTher approach is to estimate the soil thermal response to climate change throughout the permafrost region. For this, we use a set of terrestrial models that are participating in the Permafrost Carbon Network Model Intercomparison Project (PCN-MIP) [35]. A set of models were forced by a combination of reanalysis data for an initial spin-up and historical period followed by a common climate anomaly applied to the historical reanalysis data for future scenarios (table 2). For each model, future climates were calculated by applying climate anomalies of the CCSM4 climate model of a future relative to a historical climate scenario, for two climate scenarios. The first scenario, RCP4.5, is a mid-range emissions pathway that reaches plateau CO_2_ concentrations by mid-century and stabilizes at 540 ppm; the second, RCP8.5, is an unmitigated ‘business as usual’ emission scenario with continuously increasing emissions and CO_2_ concentrations that reach 935 ppm by 2100. One model (UW-VIC) reports only the RCP4.5 scenario. Though many of these models also include C and other biogeochemical cycles, we do not use these in this analysis. A separate analysis on the C dynamics of these models is underway [35].

**Table 2.** List of models used for soil thermal calculations, as well as key aspects of the models and what atmospheric conditions were specified as their current-climate upper boundary conditions.

model name	key reference	no. soil layers	maximum soil depth (m)	organic soils included?	reanalysis forcing
CLM4.5	[36]	30	45.1	yes	CRU-NCEP
GIPL2	[37,38]	300	200	yes	CRU-NCEP
JULES v3.4.1	[39]	16	20.8	no	WATCH
ORCHIDEE-MICTV3	[40,41]	32	47.4	yes	WFDEI (1978–2009)
SiBCASA	[42,43]	25	15.0	yes	CRU-NCEP
TEM6	[44]	8	36 m, but reports	yes	CRU-NCEP
only to 3 m here		
UVic	[45]	14	250	no	CRU-NCEP
UW-VIC	[46]	25	26.7	no	[47]

For each model, we take soil temperatures over the period 2010–2100, and calculate the total fractional C loss for each layer, soil horizon type and C pool, following equation (2.1). Inputs to the active layer and outputs as functions of soil temperature are as described above and inputs are here assumed to be independent of temperature, as our goal is to identify the response of soil C decomposition to warming in the absence of changes to productivity. We prognose the C balance by taking the monthly mean-soil temperatures, evaluating the temperature function for each month to calculate monthly *k* values, average the *k* values over the year and then explicitly integrate equation (2.1) with a time step of 0.1 years. The annual time-averaging of monthly *k* values rather than monthly temperature is necessary because of the strongly nonlinear relation between *k* and temperature. Note that, for calculations of transient deep C change, we exclude one soil temperature model (TEM6) from the Yedoma and thermokarst calculations because it does not report soil temperatures below 3 m, and instead we add the mean of all other model estimates of deep soil C losses to the shallow soil C losses.

### (f) Calculation of carbon–climate feedback parameter

The carbon–climate feedback factor, *γ*, as calculated in the absence of CO_2_ fertilization, is simply the ratio of the total change in area-integrated ecosystem C to the global temperature change. To calculate a feedback factor for the permafrost zone, *γ*_P_, we need to normalize the C losses by the degree of global warming,

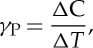
where ΔC is the total change in soil C and Δ*T* is the total change in global temperature. Global temperature change is used as the denominator for this and all other climate feedback terms so that they can be compared directly; however, the normalization by the amount of global rather than regional warming introduces a degree of dependence on the Arctic amplification of the climate model used to drive the land-surface models. Because we are holding vegetation processes constant here, the feedback term only includes changes to the soil and permafrost pools.

## 3. Results

### (a) Initial soil C distributions

Soil C distributions, as disaggregated by suborder and horizon type, are shown in figure 1. As has been pointed out previously (e.g. Harden *et al*. [16]), the major reservoirs of soil C in permafrost soils are in two main horizon type–suborder combinations: organic layers of Histel soils and mineral layers of Turbel soils. Orthel soils also contain substantial amounts of C in mineral horizons, and occupy a warmer climate space that is more peripheral to the permafrost zone than the Turbel soils; 1–2 m and 2–3 m disaggregated soil maps are not shown but show similar patterns to the 0–1 m soil maps. The deeper Yedoma and thermokarst C deposits are much less widespread and have mostly lower C concentrations than soils in the 0–3 m depth interval, but in places where they occur they can be very thick, leading to large C stocks [23], particularly in the thermokarst deposits [9].

### (b) Soil thermal dynamics

**Figure 2.**
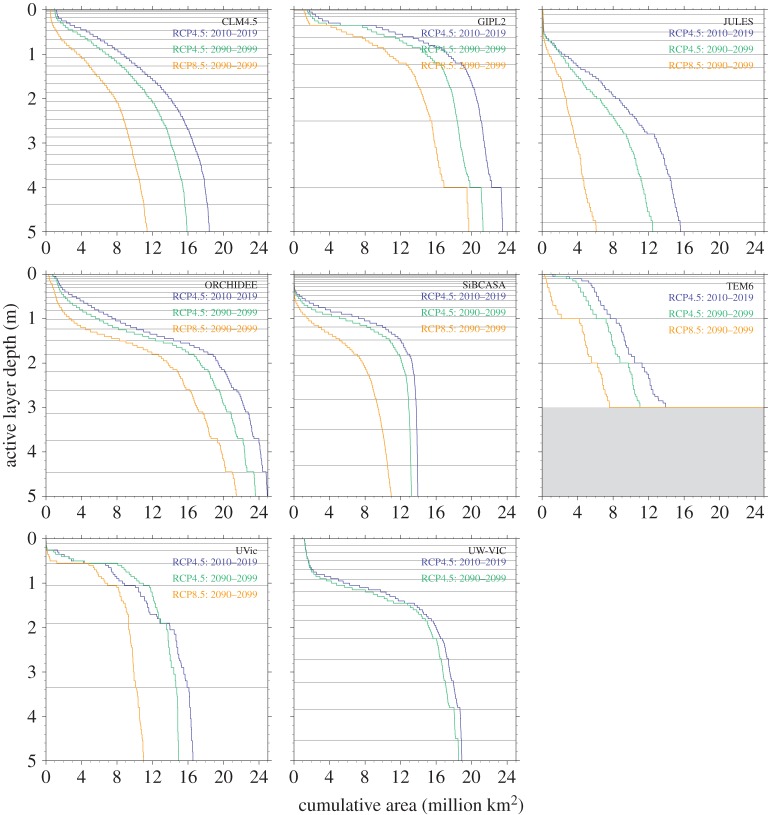
Cumulative distributions of permafrost active layer thicknesses for each of the models used in driving the soil temperature fields in PInc-PanTher, for current and end-of-century climates under moderate (RCP4.5) and high (RCP8.5) warming scenarios as driven by CCSM4 climate anomalies. Horizontal lines show the edges of model vertical levels for most models; exceptions are for GIPL2 and TEM6, which have many more levels than shown for internal calculations but output soil temperature at only the centres of the levels shown. In most models, permafrost areas decrease at all depths with warming.

The soil temperature fields calculated by the land-surface models show a range of initial permafrost areas as well as active layer thickness distributions (figure 2). All of the models used here have soil thickness deeper than shown here, although one model reports soil thermal dynamics down to only 3 m. Initial active layer thickness differs among models and includes models that have fairly shallow active layers (e.g. GIPL2 and SiBCASA), intermediate active layer thicknesses (e.g. CLM4.5) and some with thicker active layers (e.g. UW-VIC and JULES).

The response of permafrost area and active layer thickness of the land-surface models to the imposed warming treatment is almost uniformly a reduction in permafrost area and deepening of active layers. The one exception is the UVic model, in which shallow permafrost areas actually increase slightly under a warming climate. This appears to be due to increased soil wetness accompanying warming, which leads to higher soil heat capacities, cooler summertime soil temperatures and shallower active layers in the regions where soils moisten. Overall, the models show a range of responsiveness of permafrost loss to warming, with some models losing almost all near-surface permafrost by 2100 under the RCP8.5 scenario (e.g. JULES). As compared with the set of CMIP5 models [20], a major difference with these models is the better agreement with current permafrost areas, with fewer outliers that show unrealistically large or small permafrost areas and active layer thicknesses in the current climate, though this is partially because of the fewer number of models participating in the intercomparison.

### (c) C inputs required to satisfy initial steady state

As discussed above, in order to satisfy an initial balance in the C cycle, a set of time-constant inputs must be specified that match the current-climate respiration losses from each SOM C pool. As the respiration losses are a function of the current soil temperatures, inputs must be specific to each model, and are also specific to the imposed initial C pool distributions (e.g. the C : N and mean pool distributions). Note that soil temperatures have already warmed relative to the preindustrial, but for simplicity we use current temperatures as those under which soils are in steady state. Initial soil C inputs (using the mean pool distributions) range from 3.6 to 8.9 Pg C yr^−1^ integrated over the permafrost area, or from 361 to 884 g m^−2^ yr^−1^ averaged over the 10.1 million km^2^ of Gelisol soil area [8] used for this analysis. The higher inputs come from the models with substantially deeper initial active layers (UW-VIC and JULES), with the majority of models requiring inputs of less than 500 g C m^−2^ yr^−1^. Given that these inputs will be a substantial fraction of net primary productivity (NPP), though necessarily smaller than NPP because of losses via fire, dissolved organic carbon leaching, herbivory, photooxidation or respiration of C prior to it reaching the stage where it would be considered an organic soil horizon, a comparison against panarctic NPP estimates is a useful upper boundary on these inputs.

McGuire *et al.* [35] report a range of 6.5–10.9 Pg C yr^−1^ for the modelled NPP during the historical period for the C cycle simulations of these models. However, this includes a much larger area (30.1 million km^2^) than the Gelisol area used here, so the per-unit land area input fluxes average 215–362 g m^−2^ yr^−1^ for the full ecosystem model estimates. The correspondence between these estimates is imperfect as they cover different areas across steep productivity gradients, and as the Gelisol area excludes areas of bare ground and inland water that are included in the area of the full model domains. Nonetheless, the comparison suggests that at least the upper range of productivity required to meet steady state in this approach is higher than probably exists, particularly as losses by non-respiratory processes discussed above mean that the litter inputs should be smaller than NPP. Because the PInc-PanTher approach requires higher initial C inputs than are likely to occur when forced by modelled soil temperatures that have deeper active layers, the comparison suggests that either these deeper active layers or some of the assumptions built into the PInc-PanTher approach are unrealistic. These assumptions include high C stocks as obtained from soil C maps, decomposability as estimated by incubations, initial steady state of the C cycle, and anoxic conditions limited to Histel soils. Decomposition from permafrost soils is slow [48]; however, the inference of long-term dynamics from the short-term incubations is difficult and remains a possible source of bias. Furthermore, the partitioning of anoxia into either fully anoxic Histel soils or fully oxic Turbel and Orthel soils is another possible source of bias, in that seasonal anoxia of unfrozen layers may be pervasive in the region. Lastly, we note that there is not a clear relationship between the initial C inputs required to meet initial steady state for a given soil temperature field and the actual C response to climate change.

### (d) Permafrost C response to warming and carbon–climate feedback estimates

**Figure 3.**
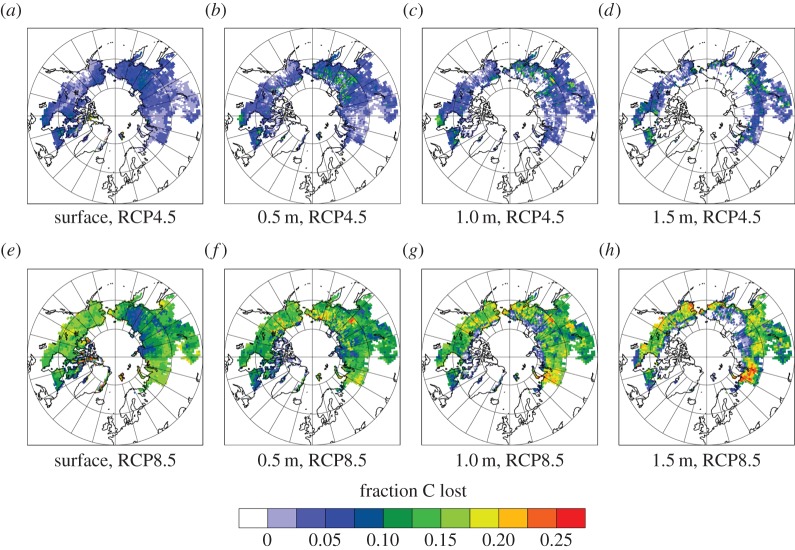
(*a*–*h*) Maps of fractional C losses over the period 2010–2100 calculated by the PInc-PanTher scaling approach at four depths (surface=1 cm, 0.5 m, 1.0 m and 1.5 m) and two warming scenarios (RCP4.5 and RCP8.5) using CLM4.5 soil temperatures as an example driving soil climate dataset. Losses are fairly uniform at the surface because of widespread lengthening of the unfrozen decomposing season and summertime soil warming; at depth C losses are zero in the area that remains permafrost and greatest at the margins of the permafrost zone where thaw leads to permanently unfrozen ground that allows continuous decomposition.

The imposed climatic warming used in the PInc-PanTher scaling approach leads to widespread soil C losses in all but one case. Projected soil losses follow a consistent pattern, similar to what is shown for one model in figure 3. In surface soils, fractional losses are fairly uniform, and do not show a strong control by permafrost distributions. The reason for this is that shallow (0–0.5 m) soils are generally already seasonally thawed and remain so with warming, such that C losses arise from a combination of (i) a longer period of unfrozen time in which decomposition can occur and (ii) warmer summertime soil temperatures that are more conducive to decomposition. Looking deeper into the soils, a clearer control by permafrost is evident, with no decomposition in the colder core permafrost areas that do not thaw even under the warming treatment versus larger losses at the southern permafrost edges, where thaw leads to a transition from permanently frozen to permanently unfrozen talik layers. As a result, the largest fractional losses are from these deeper permafrost soils along the southern permafrost boundary, which thaw early and then stay thawed for the duration of the scenario.

**Figure 4.**
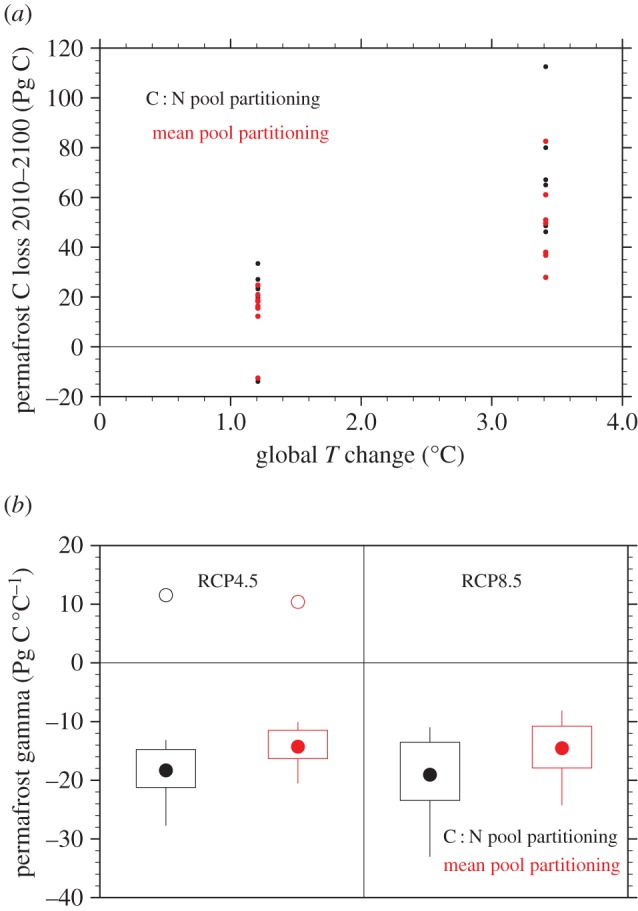
(*a*) Total integrated C losses using the PInc-PanTher scaling approach for interval 2010–2100 for two warming scenarios, two different assumptions of initial C pool partitioning and multiple soil temperature models. (*b*) Permafrost carbon–climate feedback parameter *γ*_P_. Sign convention for *γ*_P_ is that negative values indicate a loss of C to the atmosphere. Open circles are outliers.

Integrated changes in C stocks over the region (figure 4*a*) show a fairly consistent response of loss that increases with the degree of warming. The models lose C in the range of 12.2–33.4 Pg C (mean 20.8, excluding negative outliers from one model) under the moderate warming (1.2°C, globally, in the period 2010–2100) of the RCP4.5 scenario. Under the larger warming (3.4°C global) of the RCP8.5 scenario, larger losses range from 27.9 to 112.6 (mean 57.4) Pg C. These losses are within the lower to central part of the range of the 37–174 Pg C (mean 92 Pg C) reported to 2100 by [11]. Using the two different assumptions of initial pool distributions discussed above, the mean pool distribution is consistently only 74–80% as large as the C : N pool distribution under both warming scenarios. The exception to this pattern is when the PInc-PanTher approach is driven by the UVic model soil temperatures, in which case it actually gains C over the twenty-first century. The reason for this is that, as discussed above, shallow soil temperatures decrease in this model over much of the domain, and lead to an increase in the shallow permafrost area, due to soil moistening that cools summertime soil temperatures.

The calculated carbon–climate feedback parameter *γ*_P_ shows a consistent pattern (figure 4*b*), with broad overlap of the distributions between the two warming scenarios, and a consistent offset of the results depending on the assumptions used in the initial C pool distributions. If we exclude the net C gain runs as outliers, mean values of *γ*_P_ for each scenario are −19.1 to −19.5 Pg C °C^−1^ for the C : N pool distribution, and −14.5 to −14.9 Pg C °C^−1^ for the mean pool distribution. Similarly, median values range from −18.3 to −19.0 Pg C °C^−1^ for the C : N pool and −14.3 to −14.5 Pg C °C^−1^ for the mean pool distributions.

Breaking down the contributions by depth increments in the upper 3 m, we calculate, for the 0–1 m interval 10.4±8 Pg for RCP4.5 and 32±10 Pg for RCP8.5; for the 1–2 m interval 3.4±4 Pg for RCP4.5 and 13±7 Pg for RCP8.5; and for the 2–3 m interval 1.9±1 Pg for RCP4.5 and 7.4±4 Pg for RCP8.5. Thus, although local emissions are high in areas of retreating permafrost, the more widespread response in shallow soils leads to a larger magnitude of losses from the shallow layers. The bulk of emissions arise from areas that already have some seasonal thaw in the current period; restricting emissions only to initially permafrost layers leads to losses of 0.9±0.5 Pg for RCP4.5 and 3.4±2 Pg for RCP8.5. Thus, at least on the time scale assessed here, the larger contribution is from warmer temperatures and a lengthened thawed period in the active layer soils rather than a deepening of active layer into permafrost layers.

The majority of calculated emissions for the period 2010–2100 come from surface soils less than 3 m deep, with a mean of 2.1 and maximum of 16.8 Pg C arising from deeper Yedoma and thermokarst deposits below 3 m. The reason for the lack of response from deposits deeper than 3 m is that most of these areas do not thaw within the timeframe and model scenarios used to force PInc-PanTher, and furthermore, as the focus of this approach is to examine a simplified, large-scale representation of C cycle dynamics in response to warming, we do not include the kind of fine-scale but potentially widespread thermokarst processes that may give rise to more rapid thaw and subsequent C losses from the Yedoma region. Thus, our results are qualitatively different from approaches that do include a parametrization of subgrid-scale thermokarst processes, e.g. Schneider von Deimling *et al*. [49], who calculate much larger C emissions from these deeper sediments.

### (e) CH_4_ flux estimates

**Figure 5.**
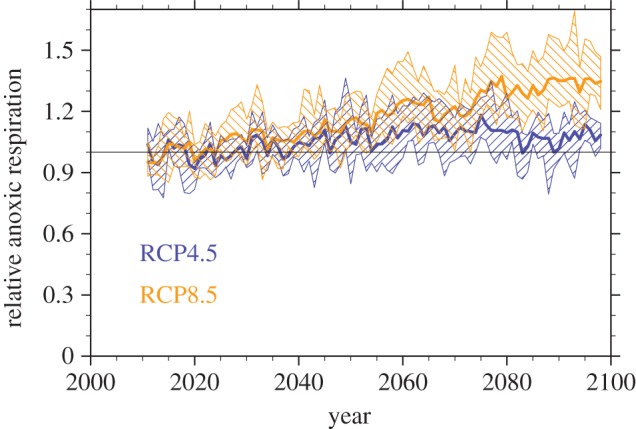
Changes relative to present of anoxic respiration rates for inferring changes to CH_4_ fluxes from permafrost C. Thick lines show ensemble mean values and hatched areas show the range across the ensemble of calculations across each of the soil temperature models. Each model trajectory is normalized to have an initial value of 1 averaged over the first decade of the simulation (2010–2020).

Aside from net C emissions, a key question of climate change feedbacks from the permafrost zone is whether potential CH_4_ emissions will also increase substantially. As discussed above, the modelled CH_4_ fluxes are assumed to be proportional to overall anoxic respiration rates (i.e. respiration arising from flooded Histel soils), and are shown in figure 5 as relative changes for both scenarios to the initial total respiration rates. Averaging over the final decade of the century, anoxic respiration rates and, therefore, CH_4_ emissions are projected to increase by 7% and 35% for the RCP4.5 and RCP8.5 scenarios, respectively. The absolute change in CH_4_ emissions depends on the mean initial CH_4_ fluxes integrated over the permafrost region. Current estimates of permafrost area wetland CH_4_ emissions range from 15 to 40 Tg CH_4_ yr^−1^ ([33], summing estimates of boreal Eurasia and boreal North America wetland CH_4_ sources). The large uncertainty on this estimate implicitly includes uncertainty in both wetland extent and CH_4_ flux densities per unit wetland area. The range of initial anoxic respiration rates from the PInc-PanTher approach is 387–1284 Tg C yr^−1^, so if we scale this to a central estimate of 30 Tg C yr^−1^ integrated source of CH_4_, this would imply that the CH_4_ flux to total anoxic respiration ratio would range from 2.4% to 7.7%, which is within the range of incubation CH_4_ production results found in Treat *et al*. [19] as well as the range of field chamber observations found in Olefeldt *et al*. [30].

Multiplying the PInc-PanTher fractional respiration changes by the range of current integrated CH_4_ emissions results in a total increase of CH_4_ emissions between 2010 and 2100 of 1.1–2.8 Tg CH_4_ yr^−1^ and 5.3–14 Tg CH_4_ yr^−1^ for the two scenarios. We can compare these against CO_2_ fluxes using a 100 year global warming potential (GWP) of 34 [50]. Because GWP has units of tons CH_4_/tons CO_2_, it therefore requires a molar mass correction to compare with total C losses, so that a GWP of 34 means that CH_4_ warms only 12.4 times as effectively as CO_2_ per unit soil C lost. This gives a 100 year warming equivalent for the changed CH_4_ emissions of 13.6–34.7 Tg C yr^−1^ to 65.7–173 Tg C yr^−1^ lost as CO_2_. Averaging the total net C losses over the 90 years of the 2010–2100 interval, the corresponding mean CO_2_-only fluxes for each scenario are 231 Tg C yr^−1^ and 638 Tg C yr^−1^ for the RCP4.5 and RCP8.5, respectively. Therefore, taking mean estimates of the relative flux magnitudes, CH_4_ emissions add another 10–18% to the 100 year radiative forcing beyond the CO_2_ emissions.

## 4. Discussion

Our upscaling approach here is essentially to construct an offline soil C cycle model of permafrost soils that is as tightly constrained by observational data as possible. As such it differs from more traditional ecosystem models in important ways. For one, we consider only one aspect of the terrestrial C cycle: the soil C budget. For the sake of simplicity we attempt to estimate vegetation C inputs through an initial steady-state assumption and hold these inputs fixed. This allows us to focus on the soil dynamics themselves, and better understand how the different approaches to handling decomposition may lead to different responses in the carbon–climate feedbacks from these soils. Because we do not include changing vegetation or inputs, this cannot be seen as an ecosystem C feedback, but only that aspect of the carbon–climate feedback that is due to the direct response of soil C to changing soil temperatures. Although vegetation is likely to change, its effect on the C budget is unclear as increased soil C losses from priming effects (changes in microbial activity and decomposition rates due to increased inputs to the soil) may counteract increased C inputs [51].

A key aspect of the PInc-PanTher decomposition trajectories, which can be seen in figure 3, are the divergent results between surface soils, where decomposition increases are moderate and more geographically uniform, versus deeper soils, where fractional decomposition losses are highest at the retreating permafrost margins and zero in the areas that remain permafrost. Overall, such a pattern leads to losses that are primarily from shallower soils. A crucial question is whether this is a realistic signature of permafrost C losses, i.e. whether C losses are in fact faster in thawed soil layers (taliks) that do not seasonally refreeze than in surface soils that still freeze seasonally even with warming, and whether a large contribution comes from lengthed thaw periods in shallow soils. Experimental and field observations of the C dynamics of retreating permafrost are needed to address this. On longer time scales, we may expect the contribution from deeper soils and initially permafrost layers to overtake shallower soils in importance, as suggested by the increasing role of deep soils beyond 2100 in Koven *et al*. [52].

For all scenarios here, we used the CCSM4 model as the atmospheric forcing for future climate anomalies. CCSM4 has an Arctic amplification (the ratio of high latitude to global surface temperature change) of 1.7, which is relatively low in the CMIP5 ensemble, in which the Arctic amplifications range from 1.5 to 2.8 [20]. Climate models that have higher Arctic amplification should in principle have higher permafrost loss rates per unit global temperature change, and so it would be useful to use a wider set of coupled land–atmosphere–ocean climate models to drive the soil thermal dynamics rather than the offline models used here, but a simple estimate is that the relatively low Arctic amplification here may lead us to underestimate the reported feedback factors by up to 65%. Alternatively, one could specify the permafrost feedback as relative to the high-latitude terrestrial temperature change, given that much of the uncertainty in the Arctic amplification is due to atmospheric processes and so not amenable to offline analysis, although that does not permit a direct comparison between permafrost and other feedbacks in the Earth system. In these experiments, the mean high-latitude temperature change, defined as the near-surface air temperature change averaged over all non-ice-covered land area north of 60° N, was 1.7 °C (RCP4.5) and 6.8 °C (RCP8.5) for the period 2010–2100.

The magnitude of the permafrost carbon–climate feedback (*γ*_P_) calculated here, in the range of −14 to −19 Pg C °C^−1^, is within the range of estimates reported in Burke *et al*. [15], though falling at the smaller end of that range. Global estimates of the terrestrial carbon–climate feedback term (*γ*_L_) from ESMs, which as discussed above have not yet included permafrost processes, are estimated to be −58.4±28.5 Pg C °C^−1^ in CMIP5 [53] and −78.6±45.8 Pg C °C^−1^ in C4MIP [1]. There is some conceptual overlap between our estimates of *γ*_P_ and the global land feedback term (*γ*_L_), which includes high-latitude soils but has not yet included representation of permafrost C processes in published intercomparisons. However, both the C4MIP and CMIP5 models show positive values of the regional feedback terms for the permafrost region [13,54], i.e. positing that high-latitude feedbacks are dominated by increased vegetation productivity with warming, and indicating that the inclusion of permafrost is a qualitatively separate contribution. Accordingly, the additional permafrost feedback contributes an additional 20–30% to current estimates of the global carbon–climate feedback over the twenty-first century. Beyond the twenty-first century, C cycle dynamics will change further, and, moreover, respiratory losses in response to twenty-first century warming itself will also continue. However, we do not extrapolate beyond this timeframe in this analysis as the fixed vegetative inputs, hydrology and other factors held constant here will become even less well justified over longer time scales. Numerical experiments with more complex models (e.g. Koven *et al*. [52]) suggest substantial nonlinearities beyond 2100.

The purpose of this paper is to follow a highly simplified scaling approach, yet there is a much broader set of processes governing ecosystem changes and carbon–climate feedbacks from high-latitude ecosystems that we are not considering in this analysis, including but not limited to: thermokarst and thermal erosion; changing vegetation productivity, distributions, decomposability and priming effects on SOM; fire; the linkages between C and nutrient cycles; changes in soil hydrology and its control of aerobic and anaerobic soil fractions; and the microbial processes responsible for decomposition and how these vary between shallow and deep soils. Our simplified scheme here may bias our results in either direction (table 3). In particular, we neglect fine-scale disturbance processes, such as thermokarst, thermal erosion and fire, which may all act to enhance CO_2_ and CH_4_ loss rates in Arctic and boreal ecosystems undergoing warming [55], which would indicate that our estimates are too low. On the other hand, we also do not include nutrient release from thawing permafrost [52], changes to vegetation productivity accompanying warming (including shrub expansion in the tundra), poleward displacement of the tundra–taiga ecotone boundary, all of which would indicate that our estimates of net C losses are too high. Furthermore, the reliance on the results of incubation studies as a fundamental pacemaker on the rate of C losses may also bias our results; the lack of fresh organic matter inputs to such incubations may result in underestimation of incubation respiration rates that would lead to an inaccurate representation of field decomposition rates [56]. Recent results highlight the significant potential effects of *in situ* priming by root exudates on SOM decomposition in permafrost soils [57].

**Table 3.** Some key processes and uncertainties not considered in this framework and their potential effect on the C feedback. (−) indicates potential for reduced net C emissions, (+) indicates potential for increased net C emissions, (±) indicates could influence net C emissions either way.

process	potential effect on the permafrost carbon–climate feedback
changing plant productivity because of warming and/or CO_2_ fertilization	increased inputs to soil: (−); potential priming effects from vegetation change on soil C turnover: (+)
biophysical effects of vegetation changes (included in some of the models used to drive soil *T* here)	decreased albedo and increased snow insulation: (+); increased shading: (−)
fire	increased fire frequency and intensity on C stocks: (+); feedbacks of fire on permafrost thaw: (+)
nutrient interactions	stimulated plant productivity with N mineralization: (−); potential priming effects from N mineralization: (+)
soil C turnover	potential biases from use of incubations, e.g. lack of fresh organic matter inputs and priming: (+)
temperature sensitivities	higher anoxic than oxic temperature sensitivities: (+); acclimation or changing carbon use efficiency: (±)
anoxia	baseline anoxia if larger than our estimate: (−); changing anoxia with warming: (±) depending on sign of change
segregated and wedge ice	slowed active layer deepening and thawing process: (−); increased vulnerability to thermokarst: (+)
CH_4_ emissions	increased productivity: (+); changed anoxia with permafrost loss: (±)
fine-scale disturbance	thermokarst and thermal erosion: (+); increased transport to watersheds and marine environment: (±)
dissolved organic C losses	if respiration increases locally: (+); if transported to deep ocean: (−)
limitation of deep C decomposition	if deep soils are microbially inhibited beyond the horizon-type changes imposed here: (−)
domain considered	inclusion of non-Gelisol soils in permafrost area: (+)
Arctic amplification of warming	if higher than our estimate: (+)

One qualitative difference between the PInc-PanTher scaling approach and the results of more complex models is the apparent linearity of C losses with increasing temperature, and resulting constancy of the feedback parameter *γ*_P_ under the two warming scenarios here, as opposed to a threshold response in the more complex models in which small amounts of climate warming lead to neutral or positive C uptake, while large temperature changes lead to C losses [42,58]. This may indicate that C losses from permafrost soils are actually more linear than the overall ecosystem C fluxes, and that while initial warming is accompanied by transient increases in vegetation uptake, in the long run these are unable to keep pace with permafrost C losses with further warming as increased productivity and vegetation C storage are unable to offset permafrost C losses. Another possibility is that there are substantial nonlinearities that we are not capturing with the simplified framework presented here. One example of such a nonlinear process is the formation of thermokarst lakes: self-reinforcing feedbacks through hydrology would enhance further thermokarst even if climate warming were halted. So the linearity we find here for top-down thaw may not apply for other types of rapid thaw that cause hydrological feedbacks.

## 5. Conclusion

We describe an approach for using soil thermal models to scale permafrost C losses accompanying warming from laboratory incubations to the panarctic, which we call the PCN Incubation-Panarctic Thermal (PInc-PanTher) scaling approach. Using a set of eight soil thermal models, soil C maps disaggregated by soil suborder and horizon type, as well as deeper deposits in some regions, and a decomposition model calibrated from a meta-analysis of permafrost incubation rates with three pools of different turnover time scales separated into three different soil horizons, we examine the response of permafrost C losses accompanying warming. Excluding an outlier model, we calculate the permafrost carbon–climate feedback parameter *γ*_P_ in the range of −14 to −19 Pg C °C^−1^ for warming during the twenty-first century, within the range of prior estimates and a globally relevant, though not dominant, contribution to the overall terrestrial carbon–climate feedback. Agreement within the ensemble of soil thermal models is fairly high, as all models show limited permafrost losses and soil warming under the RCP4.5 scenario and substantially more permafrost loss under the RCP8.5 scenario. The approach projects anoxic respiration rates—and therefore CH_4_ emissions—to increase by 7% and 35% under the RCP4.5 and RCP8.5 scenarios, respectively, at the end of the century, which will contribute to further warming, though the magnitude of this warming is substantially smaller than the magnitude of CO_2_ emissions. We propose that the PInc-PanTher approach is a useful way of identifying one aspect of C cycle changes accompanying global warming in the permafrost region, and provides an observationally constrained estimate of the likely permafrost carbon–climate feedback magnitude.

## Data accessibility

All model projections of soil temperatures used in this study are available at http://portal.nersc.gov/archive/home/c/cdkoven/www/PInc-PanTher/.
